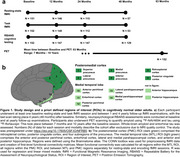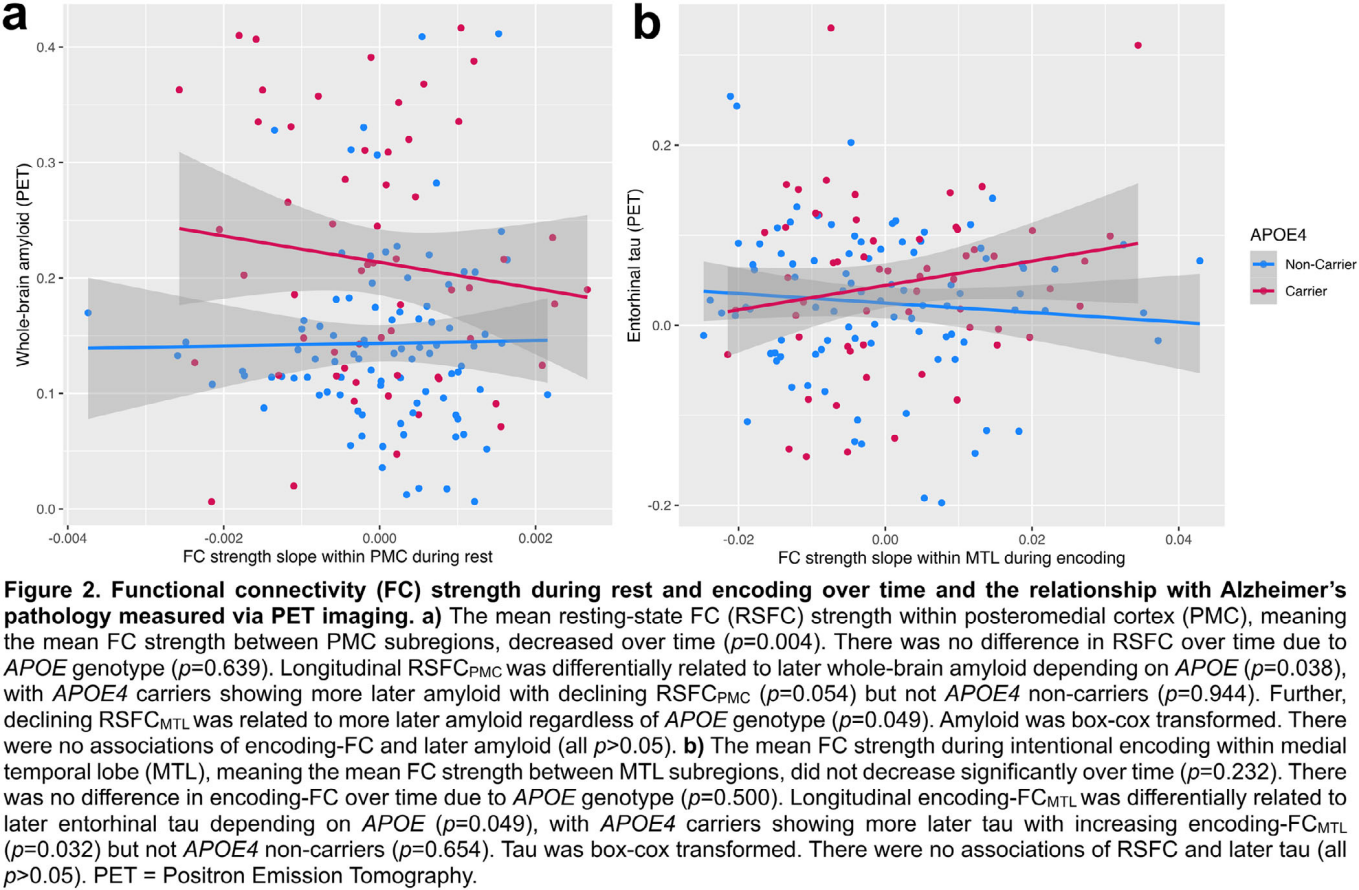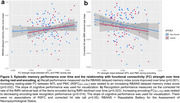# Change in functional connectivity strength during rest and encoding is differentially related to Alzheimer's pathology and memory depending on *APOE* genotype

**DOI:** 10.1002/alz70856_100192

**Published:** 2025-12-25

**Authors:** Larissa Fischer, Jenna N. Adams, Eóin N. Molloy, Jennifer Tremblay‐Mercier, Jordana Remz, Alexa Pichet Binette, Natasha Rajah, Sylvia Villeneuve, Anne Maass, PREVENT‐AD Research Group

**Affiliations:** ^1^ German Center for Neurodegenerative Diseases (DZNE), Magdeburg, Germany; ^2^ University of California, Irvine, CA, USA; ^3^ Division of Nuclear Medicine, Department of Radiology & Nuclear Medicine, Faculty of Medicine, Otto von Guericke University, Magdeburg, Germany; ^4^ Douglas Mental Health University Institute, Centre for Studies on the Prevention of Alzheimer's Disease (StoP‐AD), Montréal, QC, Canada; ^5^ Clinical Memory Research Unit, Department of Clinical Sciences Malmö, Lund University, MOntreal, QC, Canada; ^6^ Centre de recherche de l'institut universitaire de gériatrie de Montréal (CRIUGM), Montréal, QC, Canada; ^7^ Toronto Metropolitan University, Toronto, QC, Canada; ^8^ Department of Psychiatry, McGill University, Montréal, QC, Canada; ^9^ Douglas Mental Health University Institute, Montreal, QC, Canada; ^10^ Institute for Biology, Otto von Guericke University, Magdeburg, Germany

## Abstract

**Background:**

The medial temporal lobe (MTL) and posteromedial cortex (PMC) are essential for episodic memory and affected early by Alzheimer's pathology, particularly in *APOE4* carriers. Functional connectivity (FC) changes within and between MTL and PMC could be detrimental or beneficial for cognition. However, the relation of those changes to Alzheimer's pathology and memory performance is unclear and most studies assess FC only during rest. We hypothesized that increasing FC strength would be associated with higher pathology burden, especially in *APOE4* carriers.

**Method:**

In this preregistered study, we analysed longitudinal 3‐Tesla fMRI over up to 4 years and cross‐sectional amyloid‐beta and tau PET (PREVENT‐AD cohort; details in Figure 1). We assessed changes in resting‐state FC (RSFC) and task‐FC during intentional object‐location encoding within (ΔFC_PMC,_ ΔFC_MTL_) and between MTL and PMC (ΔFC_MTL‐PMC_). The sample included 152 cognitively unimpaired older adults (63±5years, 102 female, 59 *APOE4*). We investigated associations between ΔFC strength with i) pathology burden and ii) change in delayed memory (RBANS composite score and fMRI‐task object recognition), and interactions with *APOE* genotype. We used multiple regression and linear mixed models including *APOE*, age, sex and education.

**Result:**

We found ΔFC by APOE interactions predicting pathology. Specifically, declining RSFC_PMC_ (*p* = 0.038; Figure 2a) was related to more global amyloid in *APOE4* carriers only. In contrast, increasing encoding‐FC_MTL_ was related to more entorhinal tau in *APOE4* carriers only (*p* = 0.032, Figure 2b). Regarding cognition, regardless of *APOE* status, increasing encoding‐FC_PMC_ was related to decreasing RBANS (*p* = 0.018) performance and object recognition (*p* = 0.001). Finally, increasing RSFC_MTL‐PMC_ was related to increasing RBANS performance (*p* = 0.032; Figure 3a), but increasing encoding‐FC_MTL‐PMC_ was related to decreasing object recognition (*p* = 0.014; Figure 3b).

**Conclusion:**

Our study shows *APOE‐*dependent and region‐specific associations of ΔFC strength within and between episodic memory areas with pathology burden and memory performance. Notably, associations differed between RSFC and task‐FC. In *APOE4* carriers, longitudinally increasing FC or “hyperconnectivity” within MTL during encoding was related to tau in line with our hypothesis. However, in PMC, longitudinally decreasing FC during rest was related to more amyloid, indicating a disconnection in PMC regions. Our study highlights that pathology‐related network changes manifest differentially during rest and task (memory encoding).